# Psychosocial Effects of the COVID-19 Pandemic on Patients With Schizophrenia and Their Caregivers

**DOI:** 10.3389/fpsyg.2021.729793

**Published:** 2021-11-05

**Authors:** Alejandra Caqueo-Urízar, Alfonso Urzúa, Felipe Ponce-Correa, Rodrigo Ferrer

**Affiliations:** ^1^Instituto de Alta Investigación, Universidad de Tarapacá, Arica, Chile; ^2^Escuela de Psicología, Universidad Católica del Norte, Antofagasta, Chile; ^3^Escuela de Psicología y Filosofía, Universidad de Tarapacá, Arica, Chile

**Keywords:** schizophrenia, psychosocial effects, COVID-19, well-being, recovery

## Abstract

The aim of this study was to analyze the psychosocial effects of the COVID-19 pandemic on 120 patients with schizophrenia, and their caregivers (control group), in the city of Arica, northern Chile. The hypotheses of this study hold that (1) self-reports of the impact of the COVID-19 pandemic among patients and caregivers would be positively correlated, (2) caregivers would self-report a greater impact of the pandemic on their daily lives, and (3) patients infected with COVID-19 would experience lower levels of mental health improvement and higher levels of psychological distress. Hypotheses were tested using correlations, mean differences, and effect sizes (Cohen’s d). The results showed that patients with schizophrenia who had been in quarantine for almost a year showed similar levels of concern as their caregivers in the domains of health and social life. However, caregivers showed significant differences from patients in the areas of income, concern, and employment status. In addition, patients who were infected with COVID-19 showed lower levels of well-being and worse psychological recovery. The implications of the findings highlight the need to incorporate mental health interventions in the pandemic health context for caregivers of people with schizophrenia. Finally, the results suggest that Covid-19 infection has a significant effect on the recovery and psychological well-being of patients with schizophrenia.

## Introduction

After the severe acute respiratory syndrome (SARS) pandemic in 2003, significantly elevated rates of psychiatric disorders and psychological distress were observed (Mak et al., 2009). Emerging evidence shows that the COVID-19 pandemic has similarly had a negative impact on mental health (Violant-Holz et al., 2020; Solé et al., 2021). Measures to control the pandemic, have had consequences for mental health related to social isolation (Marroquín et al., 2020; Smith and Lim, 2020) and lifestyle changes (Flanagan et al., 2021). A systematic review analyzed the consequences of the COVID-19 pandemic on mental health; it found that the general population had decreased psychological well-being and higher anxiety and depression scores compared to life before COVID-19. The population with pre-existing psychiatric disorders reported a worsening of psychiatric symptoms independent of the COVID-19 contagion (Vindegaard and Benros, 2020). The current COVID-19 pandemic has had a significant impact worldwide, leading to an increased burden on patients with schizophrenia and related disorders (Kozloff et al., 2020; Yao et al., 2020), which may affect the well-being of these patients (Burrai et al., 2020). Therefore, it is plausible that this impact on mental health translates to lower levels of psychological recovery in people with pre-existing mental disorders such as schizophrenia.

People with pre-existing psychiatric disorders are a vulnerable population. They have higher risks of infection and COVID-19 complications than those without a mental disorder, both due to cognitive deficits and comorbid conditions including obesity, diabetes, and hypertension (Shinn and Viron, 2020; Yao et al., 2020). Psychiatric patients are more likely to show moderate to severe worry about their physical health due to concerns that they may have unknowingly contracted the virus. They are also less likely to use effective coping strategies to manage stress (Chua et al., 2004; Phillips et al., 2009; Colizzi et al., 2020; Solé et al., 2021). In addition, there are other factors affecting the mental health of patients with psychiatric disorders, such as delays in the delivery of psychotropic medications, lack of access to primary care or outpatient clinics, increased financial hardship, longer lengths of stay at home, and more impoverished living conditions due to shortages of basic supplies (Hao et al., 2020).

Current evidence suggests that people with schizophrenia may have an increased risk of mortality and morbidity from COVID-19, although the underlying mechanisms are unclear (Mohan et al., 2021). While schizophrenia is recognized as a public health problem in Chile, there are no recent studies on its prevalence. The latest reports have stated that prevalence of schizophrenia was between 1.4 and 4.6 persons per thousand, with an incidence of 12 new cases per hundred thousand each year, and schizophrenia and other psychoses were responsible for 1.87% of the total years of life lost due to premature death and disability in Chile (MINSAL, 2009). Chile’s outdated epidemiology of schizophrenia reveals a critical and complex invisibilization, considering that there are no official figures available which situate the panorama of schizophrenia within the current health crisis. Since the beginning of the pandemic, 1,615,771 people have been infected in Chile and the cumulative incidence rate is 9,931.1, but the current incidence rate is 60.1 (MINSAL, 2021).

Despite the COVID-19 pandemic’s enormous media presence and profound impact on society, evidence on the subject is still limited. However, the available research links social isolation and loneliness with poor mental health and increased psychological distress in the general population, highlighting a need to assess global results in order to better contextualize Chile’s situation (Burrone et al., 2020; Leiva et al., 2020). A group of researchers using self-reported data evaluated the psychological impact of fears and concerns in the general Ibero-American population, noting pervasive fear in participants during periods of isolation, particularly health-related fear (contamination, illness, and death due to coronavirus), fear related to work and loss of income, and fear of social isolation (Sandín et al., 2020). Feelings of uncertainty are also linked to the social and economic consequences of isolation measures (Johnson et al., 2020; Rodríguez-Pinzón, 2020). Other research has found that the impact of the COVID-19 pandemic on mental health is less severe for people with better psychosocial support from their family and social networks (Lei et al., 2020; Ni et al., 2020). From the above, it is possible to conclude that the psychosocial impact of COVID-19 includes increased worry, fear associated with loss of loved ones and health problems, anxiety surrounding loss of income and employment, and deteriorating mental well-being due to changes in social habits (Johnson et al., 2020; Lei et al., 2020; Ni et al., 2020; Rodríguez-Pinzón, 2020; Sandín et al., 2020).

Clinical outcomes between patients diagnosed with schizophrenia and their caregivers tend to be addressed by isolation. However, understanding the degree of family functioning, particularly its concordance and correlates between patient and primary caregiver perceptions, can serve as a platform for achieving comprehensive patient care (Hsiao et al., 2020). A recent study emphasizes that perceptions of the patient-caregiver relationship play a fundamental role in the health-related quality of life of patients with schizophrenia and their caregivers (Hsiao et al., 2021). Previous studies in northern Chile have already established that the burden and restraint of altered behavior correlate with worsened patient-caregiver relationships (Caqueo-Urízar et al., 2016), and that the quality of relationships with relatives and caregivers has a significant impact on the patient’s quality of life (Caqueo-Urízar et al., 2017). The degree of agreement in perception of patient suicidality, number of previous hospitalizations, and quality of care is often similar between patients and their primary caregiver, with patient-caregiver dyadic analysis being a good predictor of family functioning (Hsiao et al., 2020). Currently, there are no available studies which analyze perceptions in patient-caregiver relationships about the impact on their daily lives during the COVID-19 pandemic; therefore, it would be relevant to compare the degree of patient-caregiver agreement with a view of developing better psychosocial interventions (Caqueo-Urízar et al., 2016; Hsiao et al., 2020, 2021).

While the impact of COVID-19 has been particularly problematic for patients with schizophrenia, studies conducted in the early phases of the pandemic reported that patients generally showed low levels of information and concern regarding contagion, likely as a result of the anti-social behaviors and tendency toward isolation that often characterize this population (Barlati et al., 2021). In contrast, their caregivers have reported high levels of stress and burden during the pandemic (Eckardt, 2020). The COVID-19 pandemic has been a major source of stress (Zucca et al., 2021) and has resulted in a negative impact on the mental health of caregivers, especially considering that most caregivers do not seek out any mental health support as those resources typically target people living with a disorder (Gallagher and Wetherell, 2020; Alexopoulos et al., 2021). Prior to the COVID-19 pandemic, the quality of life for caregivers of people with schizophrenia was already low (Boyer et al., 2012; Stanley et al., 2017). During the pandemic, caregivers are concerned not only for their own health, but also for the continuity of care and well-being of their family member with schizophrenia (Yasuma et al., 2021). While it is quite possible that there is a similar perception of the impact of COVID-19 among patients with schizophrenia and their caregivers, the disconnect associated with schizophrenia and the greater burden on the caregiver associated with avoiding COVID-19 infection would be expected to result in a greater perceived impact from caregivers.

The clinical features of schizophrenia suggest that this population may be at higher risk of contamination, and infected patients are also expected to be at higher risk of poor outcomes or complications from COVID-19, mainly due to higher rates of comorbidity and possible immunodeficiency associated with schizophrenia (Fonseca et al., 2020; Barlati et al., 2021). A study conducted in Chile showed that participants with schizophrenia had, at some point in their lives, experienced different forms of discrimination including job discrimination, lack of social support, acts of ridicule, disqualifying acts, and social isolation, among others (Herrera, 2018). Likewise, the stigma associated with COVID-19 poses a serious threat to the lives of healthcare workers, patients, and survivors of the disease (Bagcchi, 2020). Therefore, it is possible that patients diagnosed with schizophrenia and COVID-19 experience twice the stigmatization, which can negatively affect their psychological well-being and recovery.

Another reason to expect that the COVID-19 pandemic will negatively impact the mental health of patients with schizophrenia is the loss of social support. Patients with schizophrenia usually have small, poorer quality social networks than the general population (Degnan et al., 2018), so the pandemic could significantly impact patients with schizophrenia due to mandatory social confinement and distancing, which decreases access to social support that aids in treatment of the disorder (Corrigan and Phelan, 2004; Townley et al., 2013; Degnan et al., 2018) and ability to cope with stress (Montross et al., 2005; Volavka and Citrome, 2011). Social distancing may also have a disproportionate impact on quality of life, substance use, symptoms of paranoia, and ability to maintain basic needs, given the heavy reliance of people with schizophrenia on income support and other community services that are more difficult to access, which could lead to further deterioration due to the pandemic (Hakulinen et al., 2020; Hamada and Fan, 2020; Kozloff et al., 2020). In addition, duration of confinement, lack of coping strategies, financial problems, changes in sleeping and eating patterns, and disruption of daily routines are COVID-19 factors that may increase anxiety, stress, and depression in these patients (Salari et al., 2020). Furthermore, restrictions on access to mental health services and hospitals have generated new complications, especially for patients receiving long-acting injectable antipsychotics, leading to an increased risk of relapse resulting from lower treatment adherence (Ifteni et al., 2020; Zhand and Joober, 2021). In an Italian study of residential and outpatient individuals with schizophrenia, those in outpatient treatment were four times more likely to perceive greater pandemic-related stress than those living in sheltered housing, and were two to three times more at risk for significant symptoms of anxiety and depression (Burrai et al., 2020).

Given the existing literature, there is a need to analyze the effects of the pandemic on those diagnosed with schizophrenia (Burrai et al., 2020). Thus far, the effects have not been fully delineated (Tzur Bitan et al., 2021), particularly in Latin American countries. Therefore, the present study aimed to analyze the psychosocial effects of the COVID-19 pandemic in a group of patients diagnosed with schizophrenia and their caregivers (control group) in Arica, northern Chile. The hypotheses for this study were as follows: (1) self-reports about the impact of the COVID-19 pandemic from patients and caregivers would be positively correlated, (2) caregivers would self-report a greater impact of the pandemic on their daily lives, and (3) patients infected with COVID-19 would experience lower levels of mental health improvement and higher levels of psychological distress. The findings may have implications for prevention and psychosocial intervention concerning patients with schizophrenia during the pandemic.

## Materials and Methods

### Methodological Strategy

A retrospective group comparison design with correlational scope was used.

### Ethics Statement

The study was approved by the Ethics Committee of the University of Tarapacá (18/2009) and the National Health Service of Chile. Written informed consent was obtained from the patients and their primary caregivers. The objectives of the study were explained, as well as the voluntary nature of participation. No compensation was offered for participation in the study.

### Participants

Participants were 120 patients diagnosed with schizophrenia according to the criteria of the International Classification of Diseases (ICD), 10th version [World Health Organization (WHO), 1992] and their relatives or caregivers surveyed during the months of August 2020 and May 2021 from three Centers of the Public Mental Health Service of Arica, Chile.

The mean age of participants was 40 years (SD = 13.7), of which 60% (*n* = 72) were male and 40% (*n* = 48) were female. Eighty-six percent (*n* = 104) were single, 60% (*n* = 72) reported being unemployed, and 70% (*n* = 85) were pensioned for mental disability. Twenty-seven percent (*n* = 32) reported having been infected with COVID-19 in the last 12 months.

The mean age of the caregivers was 57 years (SD = 15.5). Twenty-seven percent (*n* = 32) were men, and 73% (*n* = 88) were women, most of whom were mothers of the 120 patients. Almost all the caregivers (90%, *n* = 107) were living with the patient. Only 41% (*n* = 49) of the caregivers reported a salary as their main source of income. Twenty-seven percent (*n* = 32) reported having been infected with COVID-19 in the past 12 months.

The following inclusion criteria were defined: (1a) Patients diagnosed with Schizophrenia according to the criteria valid for the Chilean health system contained in the ICD, 10th version [World Health Organization (WHO), 1992], users of the various outpatient facilities of the Public Mental Health Service of Arica, (2a) Primary caregiver defined as the person who spends more hours per day attending and caring for the patient (Gutiérrez-Maldonado et al., 2005); and (3a) only those patient-caregiver dyads that explicitly stated their willingness to participate by signing the informed consent form were considered.

On the other hand, non-inclusion criteria were defined as: (1b) Patients with a history of neurological disorders (including epilepsy and head injury) or other diseases affecting the central nervous system (blindness, deafness); (2b) Patients with dual pathology; and (3b) Patients with a clinical history of cognitive disorders or significant intellectual deficits that hindered their understanding of the interviewer’s questions and the questionnaires used.

### Instruments

#### COVID-19 Pandemic Concerns Measurement Guideline

In light of the Coronavirus Fears Scale used by Sandín et al. (2020) and the absence of valid questionnaires for the Chilean population to assess perceptions of the COVID-19 pandemic’s impact, an *ad hoc* scale was developed based on self-reports in which participants were asked to characterize their level of exposure to COVID-19 (contagion, close contact, deceased family members, or close relatives). Subsequently, they were asked to assess their perception of the COVID-19 pandemic’s impact on main areas of their daily life such as health, general worry, job occupation, social life, and income, using a Likert scale ranging from 1 = “Not at all” to 5 = “Too much”. The level of internal consistency was assessed, delivering scores (α = 0.80) in the patient sample and (α = 0.79) in the caregiver sample. A sample of the administered instrument is provided in the Supplementary Appendix 1.

#### Kessler Psychological Distress Scale (K10)

The K10 (Andrews and Slade, 2001) was used to assess the level of anxiety and depression symptoms experienced by a person during the 4 weeks prior to participating. The scale consists of 10 items rated on a five-point scale ranging from 1 = “Not at all the time” to 5 = “All the time.” A higher score on the K10 indicates greater psychological distress. The K10 has been found to have good content validity (Brooks et al., 2006), and predictive validity for DSM-IV affective disorders (Hides et al., 2007) and serious mental illness (Kessler et al., 2003). The K10 was translated into Spanish by Aranguren (2010), and Vargas Terrez et al. (2011) examined the psychometric properties of this instrument in Chile.

#### Recovery Assessment Scale (RAS-24)

The recovery assessment scale (RAS-24) (Corrigan et al., 2004) evaluates the subjective assessment of personal recovery regarding mental health, and includes 24 items that resulted from factor analysis of the original 41-item scale. The factors that make up the scale are personal confidence and hope (9 items), willingness to ask for help (3 items), goal and success orientation (5 items), reliance on others (4 items), and no domination by symptoms (3 items). The response options are on a 5-point Likert scale (1 = “Strongly disagree” to 5 = “Strongly agree”). Currently, there is no cut-off point for interpreting RAS-24 scores; thus, in order to reduce arbitrariness, the scores were interpreted using quartiles (Q1 = 3.29; Q2 = 3.75; and Q3 = 4.21). Higher scores indicate more advanced, or better, personal psychological or mental health recovery. The RAS-24 presents adequate evidence of reliability and validity (Corrigan et al., 2004) and is probably the most widely used measure of recovery in research (Salzer and Brusilovskiy, 2014; Van Eck et al., 2018). The RAS-24 has been translated into Spanish by Muñoz et al. (2011), and Zalazar et al. (2017) examined the psychometric properties of this instrument in Argentina.

#### Positive and Negative Syndrome Scale for Schizophrenia

The positive and negative syndrome scale (PANSS) (Kay et al., 1987) is a 30-item self-report scale developed to assess psychotic symptoms in individuals with schizophrenia. There are five subscales in the PANSS that measure positive (5 items), negative (7 items), excitation (5 items), depression (4 items), and cognitive (3 items) symptom types (Lancon et al., 1998). Responses use a 7-point Likert scale (1 = “Absent” to 7 = “Extreme”). Scores are obtained by calculating the sum of all responses. The scores were interpreted according to the cut-off points of Leucht et al. (2005), where 58–74 suggests “mildly ill,” 75–94 suggests “moderately ill,” 95–115 suggests “markedly ill,” and 116 and above suggests “severely ill.” The PANSS has been translated and validated in Spain by Peralta and Cuesta (1994), and Fresán et al. (2005) examined the psychometric properties of it in Mexico.

#### Clinical and Treatment Data

Clinical variables included age at onset of the disorder (defined as the age at which the first acute psychotic episode appeared), age at onset of treatment, and the presence or absence of treatment (such as pharmacological treatment, psychotherapy, family psychoeducation, cognitive rehabilitation, and occupational therapy).

### Procedure

The present study is part of a larger project on longitudinal indicators of recovery in patients with schizophrenia. To ensure the safety of the researchers, it was necessary to create guidelines to reinforce COVID-19 contagion patterns, as well as to serve as an additional source of information to control for possible extraneous variables that could affect the recovery trajectories when gathering data on the perceptions of the patients and their caregivers, about the impact of the pandemic.

Given the legislative regulations in Chile that protect the right to medical privacy and confidentiality for users of the public health system, the researchers were only able to contact the participants and access their clinical information once the patient confirmed their willingness to participate in the study. Treatment center staff were responsible for selecting potential candidates to participate in the study, including only people diagnosed with schizophrenia and excluding patients experiencing psychotic decompensation, severe cognitive impairment, and/or intellectual disability. Once a list of potential participants had been established, the collaborating treatment center staff contacted the candidates by telephone. Patients and their caregivers who voluntarily agreed to participate in the study were asked to go to the treatment center to sign the informed consent form and complete the questionnaires according to their time availability, while respecting the social distancing protocols established by the Chilean health authority. Only patients who were receiving treatment for schizophrenia were included in the study. Patients diagnosed with schizophrenia and a comorbid disorder were not recruited.

The principal investigator hired three clinical psychologists in December 2019 to conduct the fieldwork. The team of evaluators was trained for 1 month for the correct administration of the questionnaires. During the months of March to June 2020, the researchers maintained contact with the treatment centers with the aim of establishing safe protocols and procedures to ensure adequate sanitary conditions during the evaluation for both participants and evaluators. Finally, the evaluation of the participants was carried out between August 2020 and May 2021, taking between 45 and 60 min to complete the questionnaire.

### Data Analysis

Considering the exploratory nature of the study, an effort was made to report the main clinical characteristics available, to provide information on the treatment received and the severity of psychotic symptoms. Therefore, descriptive statistical analysis was performed. The first hypothesis was tested by calculating Pearson’s correlation coefficients. The second hypothesis was tested using a paired samples *t*-test to compare differences in patients’ and caregivers’ perceptions of the impact of the pandemic on various aspects of daily life. The third hypothesis was tested using an independent samples *t*-test to compare recovery and psychological distress scores between patients with schizophrenia who reported COVID-19 infection during the past 12 months and those who did not. The effect size of the differences was estimated using the coefficient d proposed by Cohen (1988). Statistical hypothesis testing of the data analysis was performed at a significance level of 5%. All analyses were performed using Jamovi 1.6 Computer Software (The Jamovi Project, 2021).

## Results

Participant characteristics are provided in Table 1. On average, the age of onset was 21.4 years (SD = 8.4) and age of first treatment was 23.8 (SD = 8.9). All patients were taking antipsychotic medication, 29.2% were receiving psychotherapy, 17.5% were receiving occupational therapy, and 9.9% were receiving cognitive rehabilitation. Only 5.8% reported severe psychotic symptoms. Fifteen percent presented mean scores above the 75th percentile, suggesting that most reported a more advanced mental health recovery process.

**TABLE 1:** Clinical and treatment patients characteristics.

**Patients (*n* = 120)**		***M (SD)* ± range or *n* (%)**
Age of disease onset		21.4 (8.4) ± 8 – 50
Age of onset of treatment		23.8 (8.9) ± 11 – 50
Pharmacological treatment	Yes	120 (100%)
	No	0 (0%)
Psychotherapy	Yes	35 (29.2%)
	No	85 (70.8%)
Cognitive rehabilitation	Yes	13 (10.8%)
	No	107 (89.2%)
Occupational therapy	Yes	21 (17.5%)
	No	99 (82.5%)
RAS-24 total		66.7 (13.7) ± 22 – 89
PANSS categorized	Mildly ill	56 (46.7%)
	Moderately ill	39 (32.5%)
	Markedly ill	18 (15%)
	Severely ill	7 (5.8%)
PANSS total		60.2 (19.5) ± 30.0 – 111.0

*M, mean; SD, standard deviation; *n*, Number of subjects; %, effective (percentage); RAS, recovery assessment scale; and PANSS, positive and negative syndrome scale.*

Table 2 provides descriptive statistics of patients’ and caregivers’ perceptions of the impact of the COVID-19 pandemic on the five areas of daily life.

**TABLE 2:** Descriptives of the areas of concern.

		**Group**	**Income**		**Concern**		**Health**		**Social life**		**Employment status**	
Mean	*(S.D)*	Patients	2.08	(1.48)	2.46	(1.23)	1.92	(1.19)	2.15	(1.41)	1.70	(1.30)
		Caregivers	2.58	(1.45)	2.97	(1.28)	2.09	(1.26)	2.32	(1.42)	2.49	(1.68)

*Patients (*n* = 120).*

*Caregivers (*n* = 120).*

The correlation matrix (Table 3) shows that, in general, the perceptions of patients and their caregivers about the impact of the COVID-19 pandemic on daily life were significantly positively correlated with income (*r* = 0.53), concerns (*r* = 0.36), health (*r* = 0.39), social life (*r* = 0.32), and employment status (*r* = 0.27). This suggests that the perceptions of patients and their caregivers may be related.

**TABLE 3:** Correlation matrix.

	**Income (P)**	**Concerns (P)**	**Health (P)**	**Social life (P)**	**Employment status (P)**
Income (C)	*0*.0.53***	0.31***	0.27**	0.07	0.25**
Concerns (C)	0.28**	*0*.0.36***	0.22**	0.05	0.07
Health (C)	0.30***	0.27**	*0*.0.39***	0.18*	0.23**
Social life (C)	0.19*	0.10	0.15	*0*.0.32***	0.07
Employment status (C)	0.48***	0.23**	0.15	0.00	*0*.0.27**

*(P), Patient reported (*n* = 120).*

*(C), Caregiver reported (*n* = 120).*

*p < 0.05, **p < 0.01, and ***p < 0.001, one-tailed.

*Bold and italic marks correspond to patient-caregiver correlations on the same dimension of the scale.*

The results of the *t*-test for related samples (Table 4) show that there were statistically significant differences in the perceptions of patients and caregivers regarding the impact of the pandemic on areas including income (*t* = −3.75, *p* < 0.001), concerns (*t* = −3.96, *p* < 0.001), and employment status (*t* = −4.68, *p* < 0.001). Similarly, according to Cohen’s d criteria, the magnitude of the difference was moderate for the three areas (*d* = −0.35 to −0.44). In this sense, caregivers tended to perceive a greater impact of the pandemic on their daily lives compared to patients. There were no significant differences in the areas of health and social life.

**TABLE 4:** Paired samples *t*-test.

	***t* ^a^**	**df**	***p***	**Mean difference**	**SE difference**	**Effect^b^ size**
Income	−3.75	117	<0.00	−0.49	0.13	−0.35
Concerns	−3.96	117	<0.00	−0.51	0.13	−0.36
Health	−1.36	117	0.08	−0.16	0.12	−0.13
Social life	−1.12	117	0.13	−0.16	0.15	−0.10
Employment status	−4.68	115	<0.00	−0.79	0.16	−0.44

*^*a*^Student’s t.*

*^*b*^Cohen’s d.*

*Patients (*n* = 120).*

*Caregivers (*n* = 120).*

Table 5 presents the results of possible mental health repercussions associated with COVID-19 infection for the sample of patients diagnosed with schizophrenia. Those who had been infected in the last 12 months had a worse recovery process (*t* = −2.02, *p* < 0.05) and experienced more psychological distress (*t* = 2.44, *p* < 0.01). Effect size analysis indicated that the magnitude was moderate for both recovery (*d* = −0.42) and psychological distress (*d* = 0.50).

**TABLE 5:** Independent samples *t*-test.

	***t* ^a^**	**df**	***p***	**Effect size^b^**
Recovery	−2.02	58.3	0.048	−0.42
Psychological distress	2.44	58.0	0.018	0.50

*^*a*^Welch’s t.*

*^*b*^Cohen’s d.*

*Covid-19 infected patients (*n* = 32).*

*Covid-19 not infected patients (*n* = 88).*

## Discussion

The present study aimed to analyze the psychosocial effects of the COVID-19 pandemic in a group of patients with schizophrenia and their caregivers (the control group) in the city of Arica, in northern Chile.

In relation to the first hypothesis, the results showed that the perceptions of patients and caregivers about the pandemic’s impact were positively correlated. This suggests that the psychosocial effects of the pandemic would similarly affect patients diagnosed with schizophrenia, and their caregivers. It is possible that the psychosocial effects of the COVID-19 pandemic, when affecting an individual within a group, will in turn affect the rest of the group members, especially those involved in care for pre-existing disorders such as schizophrenia (Yasuma et al., 2021) and dementia (Greenberg et al., 2020; Altieri and Santangelo, 2021), or those raising children with cerebral palsy, autism, and attention-deficit/hyperactivity disorder (Dhiman et al., 2020). Overall, the evidence suggests that, during scenarios such as the COVID-19 pandemic, an increased demand for professional support combined with reduced levels of informal support can lead to serious risks for both caregivers and patients.

Although the perceptions of patients and caregivers were positively correlated, caregivers were significantly more affected than patients in the areas of income, concerns, and employment status, while no differences were observed in the areas of health and social life. It is possible that the pre-existing conditions of restricted personal freedom in people with a diagnosis of schizophrenia contributes to better adjustment to the impact of the pandemic in areas of daily life, compared to healthy people who are not accustomed to the limitations of freedom required by confinement (Burrai et al., 2020). Additionally, caregivers may be in a position of greater burden because they assume responsibility for the patient in addition to other tasks such as household management or economic support; it is expected that they would be affected to a greater extent than patients, who generally do not work and whose income is dependent on state benefits or the support of other family members. These results are consistent with the second hypothesis of this study. These findings are similarly in line with previous studies that emphasize a close relationship between patients’ and caregivers’ views that shape family functioning (Caqueo-Urízar et al., 2016; Hsiao et al., 2020, 2021). The relationship between patient and primary caregiver perspectives underscores the importance of family interventions to better address the psychosocial consequences of the COVID-19 pandemic.

In relation to the third hypothesis, the results showed that patients who had been infected with COVID-19 had higher levels of psychological distress and worse mental health recovery than those who had not been infected. This is similar to what was proposed by Fonseca et al. (2020), who reported that people with schizophrenia are a vulnerable group in the face of an infectious disease outbreak, given their high comorbidity and immunodeficiency, limited access to community care, and the risk of medication interruption that increases the risk of relapse or worse clinical outcomes. COVID-19 treatment teams may also be unprepared to treat patients with severe mental disorders. Additionally, stigma related to schizophrenia may discourage patients from seeking help. They may experience discrimination when accessing care, resulting in them being underdiagnosed for comorbid physical illnesses, being less likely to receive definitive screening and interventions, and more likely to receive poorer quality care in general (Kozloff et al., 2020). The fact that people with severe mental disorders such as schizophrenia have greater difficulty recognizing and communicating physical symptoms or health needs (Shinn and Viron, 2020) may also contribute to poorer recovery and increased psychological distress.

Although differences were observed in the levels of well-being and recovery in patients infected with COVID-19, it should be noted that the number of patients infected was small, contrary to previous study findings where these patients tended to have higher rates of infection (Kozloff et al., 2020; Moreno et al., 2020). The low infection rate can be explained by the low social contact the patients tend to have, which was increased by a prolonged quarantine of almost 1 year. It can also be explained by the fact that most of the patients were not married or did not have a partner, which may have reduced the chance of infection, as was found by Tzur Bitan et al. (2021) in Israel.

This study has a few limitations. First, the sample size was relatively small, and availability sampling was used to recruit participants. Therefore, there are limitations in the generalizability of the results. Second, at the time of the study, there were no questionnaires that assessed COVID-19 pandemic-related psychological variables, so a newly created measure assessed the impact of the pandemic on daily life. Therefore, the findings should be interpreted with caution as the measure established provides a simple and reduced view of the impact of the pandemic, in which it is clear that – despite the good levels of Cronbach’s alpha – it is an insufficient measure and requires further development. Third, there are limitations associated with the characteristics of the sample. Patient diagnosis was based on a psychiatric evaluation and ICD-10 criteria. There was no confirmation of the diagnosis through other criteria (e.g., ruling out other diagnoses through a blood test, MRI, or CT scan), and the more recent ICD-11 was not used because there was no standardization of the ICD-11 in Chile. Moreover, caregivers are likely to have some characteristics that may not be found in the general population because of their role. Therefore, the results may not be generalizable to non-caregivers. However, an advantage of including caregivers in the study is that they tend to share environmental qualities with the patients, which makes them a relevant comparison group for the purpose of this study.

Future longitudinal studies should evaluate the consequences of the pandemic not only on patient clinical outcomes, but also on their well-being and recovery, as well as the consequences in terms of caregiver burden, mental health, and well-being.

Although it is a descriptive study, this is the first study of patients with schizophrenia in Latin America that examined the psychosocial impact of the pandemic during which there was a prolonged period of quarantine.

## Conclusion

The results showed that patients with schizophrenia from northern Chile, who had been in quarantine for almost a year, showed similar levels of concern as their caregivers in the domains of health and social life; however, caregivers showed significant differences from patients in the areas of income, concern, and employment status. In addition, patients who were infected with COVID-19 showed lower levels of well-being and worse mental health recovery.

The implications of this study are related to the need to increase healthcare system support, access to mental health services, and federal economic aid, not only for patients but also for caregivers, in order to reduce poor clinical outcomes and caregiver burden.

## Data Availability Statement

The raw data supporting the conclusions of this article will be made available by the authors, without undue reservation.

## Ethics Statement

The studies involving human participants were reviewed and approved by the Ethics Committee of the University of Tarapacá and the National Health Service of Chile. The patients/participants provided their written informed consent to participate in this study.

## Author Contributions

AC-U and FP-C: conception and design of the research, preparation of the introduction of the manuscript, data collection, data analysis, and discussion of the manuscript. RF: conception and design of the research, preparation of the introduction of the manuscript, and discussion of the manuscript. AU: preparation of the introduction of the manuscript, data collection, and discussion of the manuscript. All authors contributed to the manuscript and approved the submitted version.

## Conflict of Interest

The authors declare that the research was conducted in the absence of any commercial or financial relationships that could be construed as a potential conflict of interest.

## Publisher’s Note

All claims expressed in this article are solely those of the authors and do not necessarily represent those of their affiliated organizations, or those of the publisher, the editors and the reviewers. Any product that may be evaluated in this article, or claim that may be made by its manufacturer, is not guaranteed or endorsed by the publisher.
