# Supplementary material for: Psychosocial Effects of the COVID-19 Pandemic on Patients With Schizophrenia and Their Caregivers
Source: Front Psychol. 2021 Nov 5;12:729793. doi: 10.3389/fpsyg.2021.729793 (PMC8602112; doi:10.3389/fpsyg.2021.729793)
Supplement: Supplementary file 1 [file Data_Sheet_1.docx]

| **Appendix 1**  **COVID-19 Pandemic Concerns Measurement Guideline**   \| **SAFETY Questions** \| ***YES*** \| ***NO*** \|  \| \| \| \| --- \| --- \| --- \| --- \| --- \| --- \| \| Do you have a history of Covid-19 infection? \|  \|  \|  \|  \|  \| \| Do you have any family members or close relatives infected with Covid-19? \|  \|  \|  \|  \|  \| \| Have you had contact with a confirmed case of Covid-19? \|  \|  \|  \|  \|  \| \| Do you have any family members or close associates who have died from Covid-19? \|  \|  \|  \|  \|  \| \| Are you concerned that your health condition may worsen due to the Covid-19 pandemic? \|  \|  \|  \|  \|  \| \| Do you consider yourself at risk for Covid-19? \|  \|  \|  \|  \|  \| \|  \| ***Not at all*** \| ***Slight*** \| ***Moderate*** \| ***More than moderate*** \| ***Too much*** \| \| *Indicate the degree to which the Covid-19 Pandemic (disease, shelter measures, etc.) has affected your daily life.* \| 1 \| 2 \| 3 \| 4 \| 5 \| \| Income \|  \|  \|  \|  \|  \| \| Concerns \|  \|  \|  \|  \|  \| \| General health \|  \|  \|  \|  \|  \| \| Employment Status \|  \|  \|  \|  \|  \| \| Social Life \|  \|  \|  \|  \|  \| |
| --- | --- | --- | --- | --- | --- | --- | --- | --- | --- | --- | --- | --- | --- | --- | --- | --- | --- | --- | --- | --- | --- | --- | --- | --- | --- | --- | --- | --- | --- | --- | --- | --- | --- | --- | --- | --- | --- | --- | --- | --- | --- | --- | --- | --- | --- | --- | --- | --- | --- | --- | --- | --- | --- | --- | --- | --- | --- | --- | --- | --- | --- | --- | --- | --- | --- | --- | --- | --- | --- | --- | --- | --- | --- | --- | --- | --- | --- | --- | --- | --- | --- | --- | --- | --- |
